# An in vivo approach for revealing physiological properties of human scalp microbiome

**DOI:** 10.1111/jocd.16524

**Published:** 2024-08-21

**Authors:** Jang Ho Joo, Jaeyoon Kim, Jae Young Shin, Yun‐Ho Choi, Seung‐Hyun Jun, Nae‐Gyu Kang

**Affiliations:** ^1^ R&I Institute, LG H&H Seoul Korea

Biofilms could form when fungi and bacteria adhere to surfaces by secreting a sticky substance in a high‐moisture environment. For example, we observed biofilms as slime on shower tiles, gunk in drains, and even plaque on teeth, which can cause tooth decay.^1^ The scalp surface provides a distinct microenvironment compared with other skin areas. The unique physiological conditions of the scalp include sebum content, moisture, pH, and topography. Microbial communities confer advantageous survival on surfaces.^2^ Based on this, we hypothesized that the scalp microbiome exhibits unique features related to biofilm formation for two reasons: (1) the high‐moisture condition owing to hair fibers and (2) high‐sebum production, which is favorable for the growth of *Malassezia* and *Cutibacterium*.^3^ Herein, we investigated an in vivo method for detecting biofilms on the human scalp using erythrosine solution (the detailed method is described in the Supporting Information [DataS1]). The stained red area (sRA) on the scalp corneous layer was observed owing to the red color of erythrosine (Figure 1A). The initial red intensity of the sRA was not significantly different among the subjects. After washing, most participants showed clean scalps. However, sRA was still observed in some subjects. Changes in the number of staining and washing steps in the same subject did not significantly influence the change in red intensity. Therefore, to determine the physiological characteristics of red‐stained positive and negative subjects, we further investigated the scalp physiological markers, sebum secretion rate, scalp barrier, and elasticity. Among them, the sebum production of the negative staining group measured 361 (Arbitrary Unit; a.u.), while the positive staining group had a sebum production of 640 (a.u.). This indicates that there is a positive correlation between sRA and sebum production. In the dry scalp case, however, sRA was still observed around scalp pores for two reasons: (1) structural hindrance and (2) a high amount of hyperkeratotic corneous layer and biofilm.^4^ Futhermore, we collected scalp corneocytes from subjects who stained positive for *Cutibacterium acnes* and *Staphylococcus aureus* using immunohistochemistry. Both bacteria showed aggregated features in the first layer, especially around hair pores (Figure 1B). Considering the mechanism of biofilm formation and distribution of bacteria on the scalp, control through topical treatment is needed.

**FIGURE 1:**
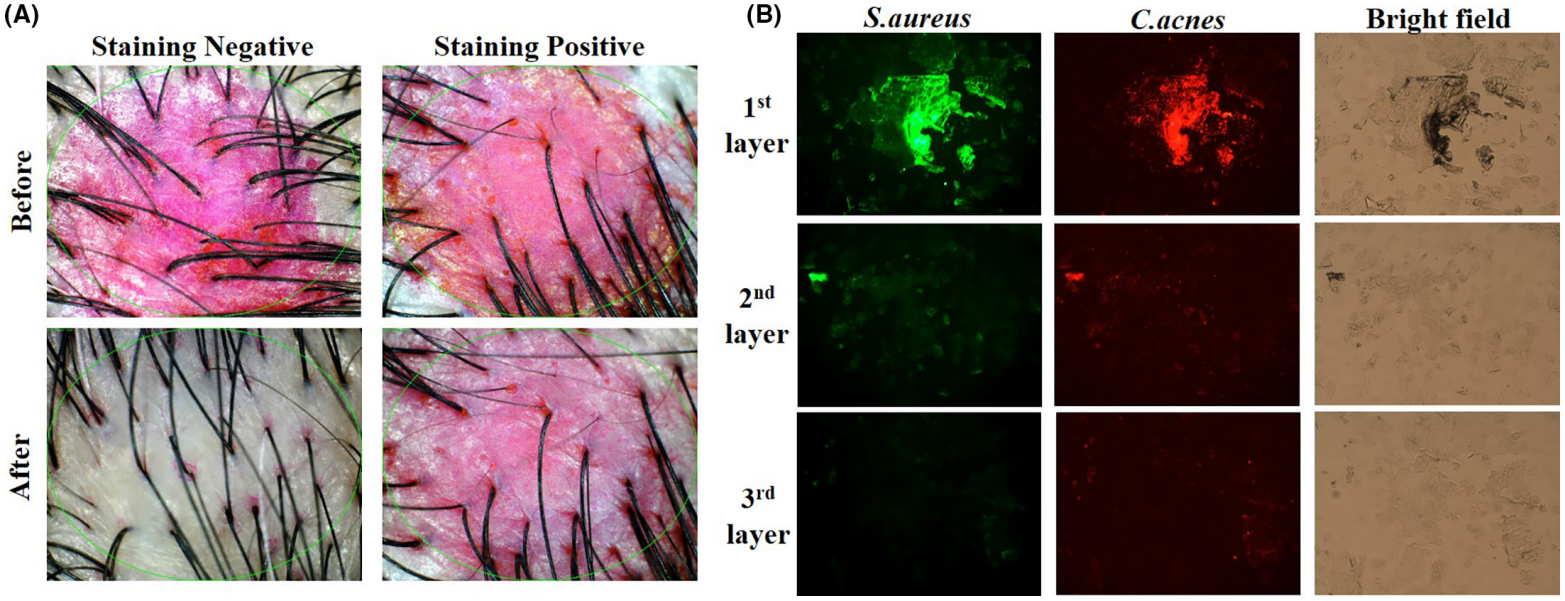
(A) Representative images of different staining types. (B) Immunohistochemistry images of tape‐stripped scalp corneocytes with *Staphylococcus aureus* (green) and *Cutibacterium acnes* (red).

Therefore, we used a scalp care product containing climbazole because it exhibits very strong antifungal/ antibacterial activities against *Malassezia*, *C. acnes*, and *S. aureus*.^5^ As shown in Figure 2, we administrated a wash‐off scalp care product containing 0.3% climbazole and 0.5% gluconolactone to reduce sRA of the scalp stratum corneum without causing chemical/physical damage to the scalp. After 2 weeks of using the scalp care product, 72% of the subjects (*N* = 26) showed that sRA was significantly reduced compared with before use. However, 27% of the participants (*N* = 10) still showed signs of sRA that were not easily removed. Consequently, we investigated hexamidine diisethionate, which reported as an anti‐microbial agent, especially on skin microbiota.^6^ The subjects exhibited a healthy scalp with a lower level of sRA after a 2‐week administration of a product containing hexamidine diisethionate (0.1%). Despite the treatment, removal of sRA was limited in the severe dandruff and oily scalp group, indicating the need for further investigation into the physiological properties and microbiome of the scalp.

**FIGURE 2:**
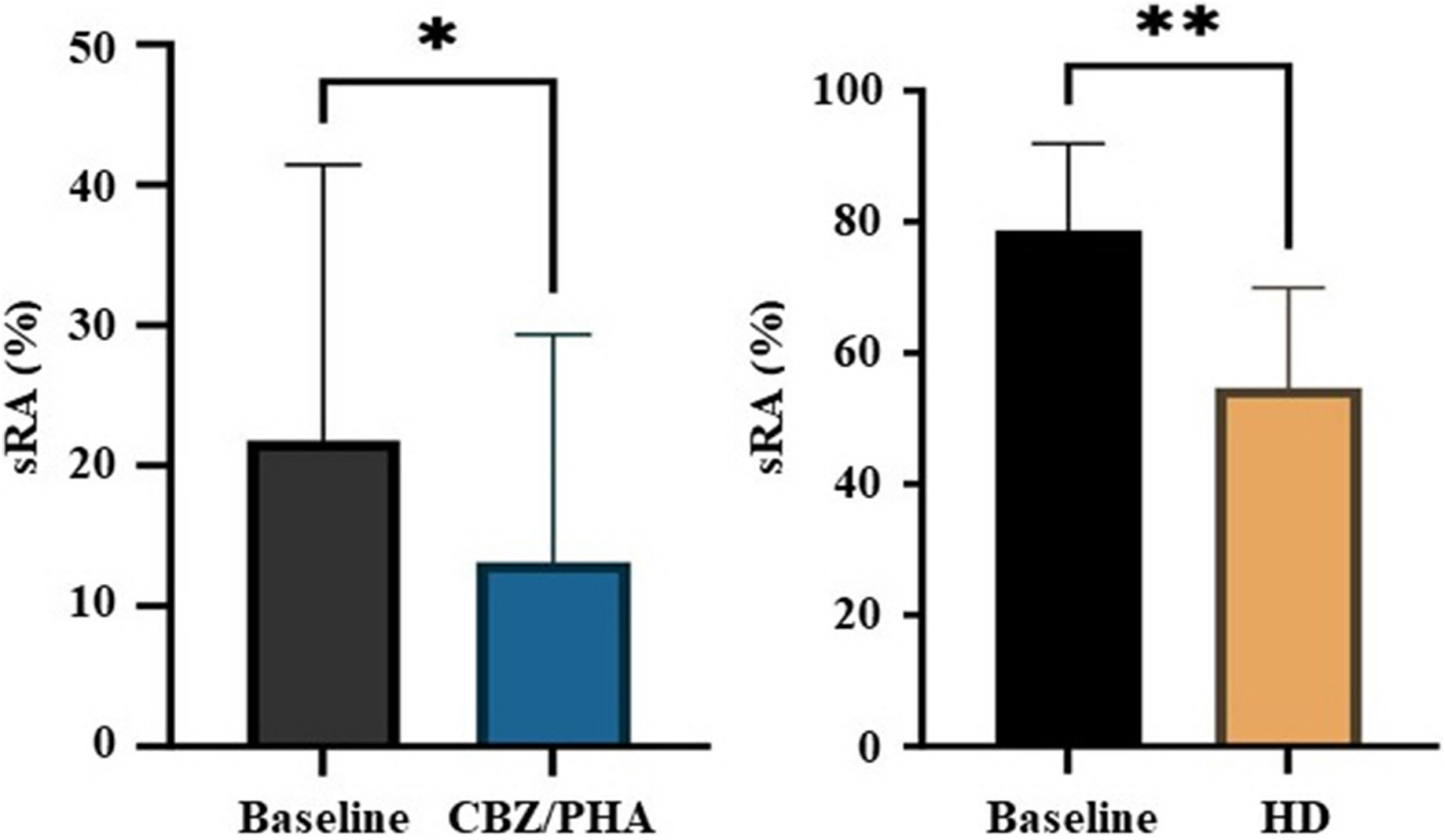
Effects of antifungal/antimicrobial agents after 2‐week administration. Stained red area (sRA) was decreased by climbazole/gluconolactone (CBZ/PHA) and hexamidine diisethionate (HD).

In this study, we developed an in vivo method for biofilm staining on the human scalp. We observed that sRA decreased in individuals treated with antifungal/microbial agents depending on the symptoms of the subject. This method could be an effective approach to examining the relationship between scalp physiology and microbiome, specifically the scalp biofilm, and help investigate new therapeutic strategies for the scalp.^7^

## AUTHOR CONTRIBUTIONS

Conceptualization: JJ, YC, NK, methodology: JJ, JK, and JS, investigation: JJ, JK, YC, writing‐original draft: JJ an JK, writing‐review and editing: JJ, JK, JS, YC, SJ and NK, supervision: NK. All authors have read and agreed to the manuscript.

## CONFLICT OF INTEREST STATEMENT

Jang Ho Joo, Jaeyoon Kim, Jae Young Shin, Yun‐Ho Choi, Seung‐Hyun Jun, Nae‐Gyu Kang were employed by the company LG Household & Healthcare Ltd.

## Supporting information


Data S1.

## Data Availability

The data that support findings of this study are available from the corresponding author upon reasonable request.
