# Supplementary material for: An in vivo approach for revealing physiological properties of human scalp microbiome
Source: J Cosmet Dermatol. 2024 Aug 21;23(12):4374–6. doi: 10.1111/jocd.16524 (PMC11626314; doi:10.1111/jocd.16524)
Supplement: Supplementary file 1 — Data S1. [file JOCD-23--s001.docx]

**Materials and Methods**

1. Subjects

The present study adhered to the tenets of the Declaration of Helsinki. The clinical studies were reviewed and approved by the Review Board of the LG Household and Healthcare Ltd. (approval number LGHH-20240314-AB-02-01). Written informed consent was obtained from all subjects prior to any study-related procedures. Volunteers with an overall healthy physical condition (47 Koreans: 30 males and 17 females) were recruited.

All the subjects washed their hair 24 hours prior to examination and did not use any anti-fungal haircare products for 2 weeks. We mainly investigated 0.3% climbazole (CBZ) and 0.1 % hexamidine diisethionate (HD) as an anti-fungal agent.

2. Analysis biofilm in human scalp

The scalp of the subjects was acclimated for 20 minutes in an air-conditioned room (temperature: 22 ± 2°C; relative humidity: 50 ± 10%). The biofilm staining was performed in midline of scalp on vertex site. First, erythrosine solution (Trace® Disclosing Solution) was dropped into a cotton swab more than 2-3 drops to be fully absorbed it. Second, the cotton swab was gently rub on human scalp surface avoiding hair to staining biofilm (staining process). Third, after 15-minute incubation, the cotton swab with water was rolled slowly to the same area of scalp by twice to remove residual erythrosine solution on skin surface (washing process). It should be noted that too much rolling force and times could damage to stained biofilm on scalp. Finally, stained area images of scalp were taken using Folliscope® (LeedM Corporation) using a 50-fold magnification lens. Obtained images were analyzed by Celleste Image Analysis Software (Invitrogen, Waltham, MA) to compare intensity of redness (Object: stained rea area, and background: unstained area color and hair color)

3. Analysis of scalp sebum level.

The participants were waiting 20 minutes in an air-conditioned room (temperature: 22 ± 2°C; relative humidity: 50 ± 10%) for acclimation. The measurements were performed in midline of scalp on the four sites; frontal, mid, vertex, and occipital site. The sebum levels were measured with a Meibometer® MB 560 (Courage-Khazaka electronic GmbH, Germany) by pressing slightly onto the scalp for 30 seconds.^1^

4. Bacteria analysis in the scalp corneocytes

Scalp corneocytes were obtained by stripping the surface of the midline of the vertex site with d D-squame® skin sampling disc (CuDerm, Dallas, TX, USA). The discs soaked with 1 mL of PBS solution (1% BSA, 5% FBS, 0.01% Tween20) for 1 hour to prevent undesired bindings between antibody and bacteria in 6-well plate. After that, the discs were washed by 1 mL of the PBS solution for 3 times, and incubated with 1.5 mL of antibody solution containing rabbit polyclonal S. aureus antibody (1:500 dilution, ab20920, Abcam, Cambridge, USA) and mouse monoclonal C. acnes antibody (1:500 dilution, D371-3, MBL, Nagoya, Japan) overnight at 4 °C followed by Goat anti-Rabbit IgG (H+L) Highly Cross-Adsorbed Secondary Antibody (1:1500 dilution, Alexa Fluor™ 488, ThermoFisher) and Goat anti-Mouse IgG (H+L) Cross-Adsorbed Secondary Antibody, (1:1500 dilution, Alexa Fluor™ 594, ThermoFisher) for 1 hour.

5. Statistical analysis

Statistical analysis was conducted using SPSS 20.0 (SPSS, IL, USA) and Prism 10.0.2 (GraphPad Software, MA, USA). Data are presented as mean ± standard error of mean (SEM). We assessed both data for normal distribution and non-normal distribution groups by Shapiro-Wilk Test. In physiological parameters following normal distribution, statistical significance (∗ P < 0.05 and ∗∗ P < 0.01) was evaluated using two-tailed unpaired Student’s t-test for comparisons between two. In non-normal distribution parameters, statistical significance (∗ P < 0.05 and ∗∗ P < 0.01) was evaluated using Mann Whitney U test with two groups. Correlation test was evaluated using Pearson Correlation test.

**Reference**

1. Yoon JS, Shim J, Lim JM, Park SG. Biophysical characteristics of dandruff‐affected scalp categorized on the basis of sebum levels. *Journal of Cosmetic Dermatology.* 2021;20(3):1002-1008.
